# Aluminum contamination of food during culinary preparation: Case study with aluminum foil and consumers’ preferences

**DOI:** 10.1002/fsn3.1204

**Published:** 2019-09-09

**Authors:** Dani Dordevic, Hana Buchtova, Simona Jancikova, Blanka Macharackova, Monika Jarosova, Tomas Vitez, Ivan Kushkevych

**Affiliations:** ^1^ Department of Plant Origin Foodstuffs Hygiene and Technology Faculty of Veterinary Hygiene and Ecology University of Veterinary and Pharmaceutical Sciences Brno Brno Czech Republic; ^2^ Department of Meat Hygiene and Technology Faculty of Veterinary Hygiene and Ecology University of Veterinary and Pharmaceutical Sciences Brno Brno Czech Republic; ^3^ Department of Gastronomy Faculty of Veterinary Hygiene and Ecology University of Veterinary and Pharmaceutical Sciences Brno Brno Czech Republic; ^4^ Centre of Region Hana for Biotechnological an Agricultural Reseach Central Laboratories and Research Support Faculty of Science Palacky University Olomouc Olomouc Czechia; ^5^ Department of Agricultural, Food and Environmental Engineering Faculty of AgriSciences Mendel University in Brno Brno Czech Republic; ^6^ Department of Experimental Biology Faculty of Science Masaryk University Masaryk Czech Republic

**Keywords:** aluminum foil, baking, consumers’ preferences, leakage, marinating

## Abstract

The aim of the work was to estimate the degree of aluminum leakage from aluminum foil during baking process of selected food/meals. The experiment included 11 different types of food (Atlantic salmon *Salmo salar,* mackerel *Scomber scombrus,* duck breasts, cheese Hermelín, tomato, paprika, Carlsbad dumplings, pork roast, pork neck, chicken breasts, and chicken thighs) baked both marinated and not marinated. The aluminum content was measured by AAS and ICP/MS methods. The highest aluminum increase was observed in the samples of marinated *Salmo salar* (41.86 ± 0.56 mg/kg), *Scomber scombrus* (49.34 ± 0.44 mg/kg), and duck breast (117.26 ± 1.37 g/kg). The research was also supported by the survey that consisted of 784 respondents with different sociodemographic characteristics. The study clearly showed the occurrence of aluminum contamination of food when it is prepared by baking in aluminum foil. It cannot be concluded that aluminum leakage will occur with each type of food. The aluminum contents found among investigated samples are not alarming, though the increase was measured up to 40 times. On the other hand, revealed aluminum contents can represent a risk for younger/smaller children and for individuals with diagnosed certain ailments.

## 1 INTRODUCTION

Aluminum is one of the most abundant elements in earth crust's mass (8% of the earth crust's mass); due to this fact, it can be explained the prevalence of aluminum content in food, though with concentrations below 5 mg/kg (EFSA, 2008; Ertl & Goessler, 2018). Daily aluminum intake depends on food types, and according to studies, following food types contribute the most to aluminum daily intake: cereals, vegetables, fruits, tea, coffee, wheat, and wheat‐based products (Bratakos, Lazou, Bratakos, & Lazos, 2012; Fekete, Vandevijvere, Bolle, & Loco, 2013; González‐Weller, Gutiérrez, Rubio, Revert, & Hardisson, 2010; Ma et al., 2016). The problem of aluminum intake can be emphasized with the fact that only small amounts of aluminum can be efficiently excreted by human bodies. Meaning that wider population can be exposed to aluminum amounts that cannot be handled by human body (Bassioni, Mohammed, Al Zubaidy, & Kobrsi, 2012).

Aluminum is used as packaging material, and it is approximately calculated that it has produced about 860 000 tons of aluminum foil per year only in the Europe (EAFA, 2016). Aluminum foil is mainly used for food packaging, cosmetics, and chemical products (approximately 75%) (EFSA, 2011). Aluminum foil is broadly used in culinary preparation of different food types due to its easiness to use, disposability, and properties such as that it transfers heat twice quicker than regular metal. It is a common culinary practice in households to wrap food in aluminum foil and baked it. These food preparation and practice can expose consumers to different aluminum amounts in dependence to food types and composition (Bassioni et al., 2012). This interaction between aluminum foil and food wrapped in it represents a potential hazardous source of aluminum in the human diet. On the other side, aluminum packaging is very popular due to its following properties: impermeability, freeze proofing, it is inert, especially it possesses a perfect dead fold characteristics and recyclability (Verissimo, Oliveira, & Gomes, 2006).

Aluminum contamination of food represents an important issue to find relationships between aluminum intake and certain serious illness such as Alzheimer's disease, Parkinson's disease, dialysis encephalopathy, bone disorder, human breast cancer, and it is also considered to be a neurotoxin; aluminum salts can be accumulated by the gut and different human tissues (bones, parathyroid, and brain). Aluminum is diversely affecting the growth rate of human brain cells. (Al Juhaiman, Al‐Shihry, & Al‐Hazimi, 2014; Al Zubaidy, Mohammad, & Bassioni, 2011; Bassioni et al., 2012; Darbre, Pugazhendhi, & Mannello, 2011; Gitelman, 1989; Kim & Clesceri, 2001; Rittirong & Saenboonruang, 2018).

The average human intake of aluminum has been calculated to be from 14 to 105 mg aluminum per week for a 70 kg adult person. The exposure of child weighing 30 kg is estimated to be from 21 to 69 mg aluminum per week. These findings are stressing out the possibility of reaching aluminum maximum tolerable weekly intake of 1mg/kg of body weight (Center for Food Safety, 2009) or 2 mg/kg of body weight (World Health Organization, 2011) (Stahl, Falk, Taschan, Boschek, & Brunn, 2018). Though, the weekly aluminum intake limit used to be 7 mg/kg of body weight (WHO, 1989).

The aim of the study consisted of two parts. The first was to evaluate the leakage of aluminum to different food types that were baked in aluminum foil. The second was to overview aluminum prevalence/contamination of food and consumers’ preferences toward the usage of aluminum foil in food culinary preparation and their awareness of harmfulness.

## 2 MATERIAL AND METHODS

### 2.1 Food samples

Atlantic salmon fresh fillet without skin (*Salmo salar*, Norway, farmed, Ocean48 s.r.o., Czech Republic), fresh whole gutted mackerel (*Scomber scombrus,* Norway, caught, FAO37 area, category of fishing gear: trolling, Ocean48 s.r.o., Czech Republic), deep frozen duck breasts with skin (Cut duck meat, quality class A, Tranzit‐Food, Kft., Nyirgelse, Hungary), cheese Hermelín (King of cheese Hermelín original, Savencia Fromage & Dairy, Czech Republic), fresh tomato (Czech Tomatoes on stem, quality class 1, Blanická s.r.o., Czech Republic), fresh paprika (Red paprika, variety California, quality class 1, EFES, spol. s.r.o., Czech Republic), Carlsbad dumplings (own preparation from raw materials according to recipe in sensory laboratory), fresh pork roast without bone, slices (Cut pork roast, Maso Uzeniny Polička, a.s., Czech Republic), fresh pork neck without bone, slices (Cut pork neck, Maso Uzeniny Polička, a.s., Czech Republic), fresh chicken breasts without skin and bone (Cut chicken breasts, quality class A, Tesco Stores ČR a.s., Czech republic), and fresh chicken thighs with skin and bone (Cut chicken thighs, quality class A, Tesco Stores ČR a.s., Czech republic). Deep frozen duck breasts were defrosted (at temperature + 2±2°C/12 hr), half of the samples were left with skin, and the skin from the second half was removed manually. Raw food samples were prepared according to commonly used recipes; marinade was added to experimental samples (the recipe composition is shown in Table 1). Each sample was packed into each of the 5 film types (area 20 × 20 cm), as well as a glass as inert packing material was also used. The baking was done during 40 min at 220ºC in the oven (Professional Ovens GARB‐IN, Model: 23 GM UMI). After cooling, the samples were homogenized, and the homogenates were vacuum packed and frozen. Analyzes were performed sequentially from thawed homogenate.

**Table 1:** Weights (g) of foodstuffs and marinade ingredients used in the research

Samples	Foodstuffs weight (g)		Marinade ingredients (g)
	NM	M	
Cheese Hermelín	120.00 ± 0.00		onion, tomato, basil, (all were fresh); olive oil, iodine salt, black pepper, sweet pepper, ground cumin, garlic granulated, chili pepper ground
Tomato	189.56 ± 22.72	192.44 ± 24.11	olive oil, iodine salt, black pepper, caster sugar
Paprika	204.79 ± 22.30	211.93 ± 21.37	olive oil, iodine salt, black pepper
Carlsbad dumplings	166.94 ± 9.77		fresh rolls, semi‐fat milk, fresh class A eggs, iodine salt, black pepper, nutmeg ground
Pork roast	93.96 ± 2.29	93.84 ± 1.82	cheese, bacon, onion; olive oil, iodine salt, black pepper
Pork neck	137.46 ± 7.30	132.39 ± 3.66	olive oil, iodine salt, black pepper, ketchup sweet, special mustard sharp
Chicken breasts without skin	163.78 ± 12.53	164.11 ± 24.25	olive oil, iodine salt, black pepper, ketchup sweet, special mustard sharp, honey, fresh basil
Chicken legs with skin	273.05 ± 21.86	273.52 ± 17.56	olive oil, iodine salt, black pepper, ketchup sweet, special mustard sharp, honey, fresh basil
Salmon without skin	98.98 ± 5.70	104.56 ± 11.48	olive oil, iodine salt, black pepper, garlic granulated, lemon juice, fresh basil
Mackerel with skin	227.55 ± 33.90	247.50 ± 31.06	olive oil, iodine salt, black pepper, garlic granulated, lemon juice
Duck breasts with skin	100.90 ± 4.71	101.89 ± 5.24	olive oil, iodine salt, black pepper
Duck breasts without skin	82.57 ± 7.97	83.46 ± 5.90	olive oil, iodine salt, black pepper

Note

Abbreviations: M, marinated; NM, not marinated.

### 2.2 Aluminum foils

Five foils present on the market were used in the research. The foils differ by purchase price, labeling (can be used for barbecue, general use in the kitchen). The following aluminum foils were used: foil A (Grill Aluminium Foil, Melitta ČR s.r.o., Praha 5, Czech Republic), foil B (Grill Alu Foil Fino, Sarantis Czech Republic, Praha 3, Czech Republic), foil C (Grand Maximo, Kaufland Czech Republic, v.o.s., Praha 6, Czech Republic), foil D (Vi‐Go! Live Good, Quickpack, Jedrzejow, Poland), and foil E (Super strong Aluminium Foil, Sarantis, Praha 3, Czech Republic).

### 2.3 Physical‐chemical analysis

The laboratory analysis included determination of pH, salt, fat, protein, and dry matter content. pH was measured using the WTW GmbH, Germany. The salt (NaCl) content was determined by titration with silver nitrate—Mohr's method (standard operating procedure, potassium chromate indicator). The lipid content was determined quantitatively (ČSN ISO 1443:1973) using Soxtec 2055 (FOSS Tecator, Höganäs, Sweden). The protein content (ČSN ISO 937:1978) was determined as the amount of organically bound nitrogen using the analyzer Kjeltec 2300 (FOSS Tecator, Höganäs, Sweden). The dry matter/moisture content was determined gravimetrically according to the Czech National Standard (ČSN ISO 1442:1997) by drying the sample to a constant weight at + 103 ± 2ºC (Binder FD 53, Germany). The dry matter/moisture values of foodstuffs were used to convert the results for the aluminum content determined in dry matter by the protocol methods (see below) to the wet mass (Tables 2, 3, 4).

**Table 2:** Aluminum contents in salmon and mackerel samples expressed in dry and wet masses (mg/kg)

Samples	Salmon (Dry mass mg/kg)	Salmon (Wet mass mg/kg)	Mackerel (Dry mass mg/kg)	Mackerel (Wet mass mg/kg)
C	0.00 ± 0.00	0.00 ± 0.00	1.17 ± 0.01	0.26 ± 0.01
CIP	0.00 ± 0.00	0.00 ± 0.00	0.00 ± 0.00	0.00 ± 0.00
C‐foil A	1.31 ± 0.02	0.54 ± 0.01	0.00 ± 0.00	0.00 ± 0.00
C‐foil B	0.00 ± 0.00	0.00 ± 0.00	1.25 ± 0.05	0.28 ± 0.01
C‐foil C	0.00 ± 0.00	0.00 ± 0.00	4.66 ± 0.10	1.14 ± 0.02
C‐foil D	0.00 ± 0.00	0.00 ± 0.00	1.35 ± 0.02	0.39 ± 0.00
C‐foil E	0.00 ± 0.00	0.00 ± 0.00	2.31 ± 0.04	0.58 ± 0.00
CM	0.00 ± 0.00	0.00 ± 0.00	0.81 ± 0.03	0.20 ± 0.00
MIP	0.00 ± 0.00	0.00 ± 0.00	0.00 ± 0.00	0.00 ± 0.00
M‐foil A	41.86 ± 0.56	20.95 ± 0.18	49.34 ± 0.44	13.42 ± 0.24
M‐foil B	35.70 ± 1.08	17.79 ± 1.14	43.69 ± 1.41	10.92 ± 0.27
M‐foil C	15.87 ± 3.62	7.95 ± 0.78	29.12 ± 0.32	7.59 ± 0.04
M‐foil D	41.62 ± 0.92	21.00 ± 0.22	15.64 ± 0.10	5.33 ± 0.02
M‐foil E	34.87 ± 0.39	15.07 ± 0.35	14.68 ± 4.33	4.56 ± 0.01

**Table 3:** Aluminum contents in duck breasts expressed in dry and wet masses (mg/kg)

Samples	Duck breasts (Dry mass mg/kg)	Duck breasts (Wet mass mg/kg)	Samples	Duck breasts—marinade (Dry mass mg/kg)	Duck breasts—marinade (Wet mass mg/kg)
C	0.00 ± 0.00	0.00 ± 0.00	CM	1.83 ± 0.04	0.52 ± 0.01
CIP	0.00 ± 0.00	0.00 ± 0.00	MIP	0.95 ± 0.01	0.40 ± 0.00
C‐foil A	1.13 ± 0.02	0.32 ± 0.00	M‐foil A	85.93 ± 6.58	41.30 ± 0.48
C‐foil B	1.11 ± 0.02	0.46 ± 0.00	M‐foil B	65.31 ± 17.40	28.78 ± 0.17
C‐foil C	1.26 ± 0.01	0.61 ± 0.01	M‐foil C	63.78 ± 0.55	25.78 ± 0.36
C‐foil D	0.00 ± 0.00	0.00 ± 0.00	M‐foil D	80.76 ± 4.01	31.73 ± 0.10
C‐foil E	0.00 ± 0.00	0.00 ± 0.00	M‐foil E	117.26 ± 1.37	45.18 ± 0.01
CS	0.00 ± 0.00	0.00 ± 0.00	CMS	0.857 ± 0.07	0.24 ± 0.00
CIPS	0.00 ± 0.00	0.00 ± 0.00	MIPS	3.96 ± 0.04	1.39 ± 0.01
CS‐foil A	0.00 ± 0.00	0.00 ± 0.00	MS‐foil A	35.69 ± 0.15	17.15 ± 0.20
CS‐foil B	0.80 ± 0.06	0.35 ± 0.00	MS‐foil B	51.71 ± 0.73	22.79 ± 0.13
CS‐foil C	0.00 ± 0.00	0.00 ± 0.00	MS‐foil C	36.31 ± 0.63	14.68 ± 0.21
CS‐foil D	1.11 ± 0.02	0.45 ± 0.01	MS‐foil D	46.72 ± 0.37	18.35 ± 0.06
CS‐foil E	0.00 ± 0.00	0.00 ± 0.00	MS‐foil E	38.39 ± 0.29	14.79 ± 0.00

** C, control; CIP, control packed in inert packaging; C‐foil A, C‐foil B, C‐foil C, C‐foil D, C‐foil E, samples packed in different foils; CM, control marinade; MIP: samples in marinade packed in inert packaging; M‐foilA, M‐foilB, M‐foilC, M‐foilD, M‐foilE, samples in marinade packed in different foils.

**Table 4:** Aluminum content in food samples—raw samples versus the highest found aluminum concentrations in the samples wrapped and baked in aluminum foil

Food samples	Raw (Dry mass mg/kg)	Raw (Wet mass mg/kg)	The highest concentration (Dry mass mg/kg)	The highest concentration (Wet mass mg/kg)	Notice^a^
Hermelín	–		4.46 ± 0.12	1.68 ± 0.02	bM foil E
Tomato	1.70 ± 0.02	0.08 ± 0.00	7.78 ± 0.18	0.37 ± 0.02	Foil A
Paprika	–		1.32 ± 0.03	0.10 ± 0.00	M foil E
Karlovarský knedlík	2.31 ± 0.02	1.07 ± 0.02	1.88 ± 0.42	0.90 ± 0.01	Foil E
Pork roast	–		15.87 ± 0.07	6.90 ± 0.08	M foil E
Pork neck	–		4.91 ± 0.06	1.74 ± 0.00	M foil E
Chicken breasts	–		5.25 ± 1.48	1.70 ± 0.04	M foil C
Chicken legs	–		3.6 ± 0.2	1.24 ± 0.03	M foil D

Abbreviations: M, marinated; NM, not marinated.

a Indication in which aluminum foil the Al concentration was the highest.

b M indicating marinated samples.

### 2.4 Aluminum content analysis

Aluminum contents were measured by two methods that included inductively coupled plasma mass spectrometry (ICP‐MS) and atomic absorption spectroscopy (AAS).

#### 2.4.1 ICP/MS protocol

Prior to the microwave digestion, the homogenized food samples were lyophilized to dryness by using a freeze dryer BETA 1–8 LD plus (Martin Christ, Germany). The dried samples (about 0.16–0.19 g) were digested in MLS 1200 Mega closed vessel digestion unit (Milestone S.r.L., Sorisole, Italy) by the mixture of 2 ml HNO_3_ and 1 ml of H_2_O_2_. The power‐controlled digestion program was applied: 2 min (250 W), 2 min (0 W), 5 min (400 W), 2 min (0 W), 2 min (500 W), 2 min (0 W), and 6 min (600 W). The digests cooled to the laboratory temperature were diluted with ultrapure water to 15.0 ml and stored at −20°C before the ICP‐MS analysis. The blank samples underwent the same working conditions as the real food samples. The quantitative analysis of aluminum was carried out using a 7700x ICP‐MS (Agilent Technologies, Tokyo, Japan) equipped with a quadrupole mass analyzer and an ASX‐520 auto‐sampler. The optimized ICP‐MS instrumental conditions for No‐Gas mode were as follows: RF power of 1550 W, plasma gas flow rate of 15.00 L/min, auxiliary gas flow rate of 0.9 L/min, nebulizer gas flow rate of 1.07 L/min and dwell time 300 ms for **^27^Al** and 100 ms for **^45^Sc** (served as an internal standard) isotopes. Each food sample was measured in six replicates.

#### 2.4.2 AAS protocol

Determination of aluminum was carried out on the ContrAA 700 instrument (Analytik Jena AG) using the ETA‐AAS electrothermal atomization method. Measurement conditions included a cuvette with a platform, wavelength 396.1520 nm, Mg (NO_3_)_2_ matrix modifier. 6H_2_O, pyrolysis temperature 1,500°C, and atomization temperature of 2,400°C. The calibration (0–100 ppb) was prepared by dilution from a standard aluminum solution (1g/L, from Analytica spol. s ro, Prague).

Microwave decomposition was performed on an ETHOS SEL instrument (Milestone, Italy). The weight of samples was approximately 1.5 g, and decomposition was done with nitric acid (67%, Analpure, Analytika spol. s.r.o.) and hydrogen peroxide (30%). Pressure decomposition took place in two stages with a maximum temperature of 200°C and a microwave force of up to 1,000 W.

### 2.5 X‐ray generator spectroscopy

The profile of each used aluminum foil was measured by using the portable X‐Ray Analyzer NITON XL3t GOLDD+ (Thermo Fisher Scientific). Before aluminum content determination, X‐Ray Analyzer was calibrated with a standard metal alloy. After successful calibration check, the analyzer was positioned into a test stand and ready for scanning. The X‐Ray Analyzer was operated in metal mode. For the metal content, all of the three beams, 30 s each, were used. First and second beams were operated at 50 keV, and third beam was operated at 15 keV.

### 2.6 The survey

The part of the research included the survey about consumers’ preferences toward the usage of aluminum foil during food culinary preparation. The questionnaires were carried out both in‐person (mainly at the Veterinary and Pharmaceutical University in Brno, Czech Republic) and online (written in Czech language with the use of Google forms/docs). The research group consisted of 784 respondents. The study was conducted between May 2018 and October 2018. The questionnaire counted 23 questions, consisting of two parts. The first part (*n* = 5) was about the demographic characteristics of respondents; the second part included questions (*n* = 18) concerning respondents’ preferences and aluminum foil usage (Table 5). More females (69.5%; *n* = 545) than males (30.5%; *n* = 239) participated in the survey, and most of them were not married (77.4%; *n* = 607).

**Table 5:** Demographic information about respondents (100%; *n* = 784)

Demographic category	Groups	Number of respondents	Percentage of respondents
Gender	Female	545	69.5
	Male	239	30.5
Marital status	Married	607	77.4
	Not married	177	22.6
Age	Under 20 years	393	50.1
	21–30 years	190	24.2
	31–50 years	153	19.5
	Over 50 years	48	6.1
Education level	Elementary school	81	10.3
	Secondary school	548	69.9
	Higher education	155	19.8

### 2.7 Statistical analysis

Statistical significance at *p* < .05 was evaluated by oneway ANOVA analysis of variance, and parametric Tukey post hoc test (in the case when Levene's test showed equal variances *p* > .05) and nonparametric Games–Howel post hoc test (in the case when Levene's test showed unequal variances *p* < .05) for finding differences within groups. Overall differences among samples were checked by principal component analysis (PCA). Additional information about the association of variables was provided by chi‐squared tests. SPSS 20 statistical software (IBM Corporation) was used.

## 3 RESULTS AND DISCUSSION

In this study, the real conditions of food culinary treatment by consumers in households were simulated to monitor the level of aluminum transfer from different aluminum foils into a food whose method of preparation, consumption size, or weight (e.g., one piece of tomato, peppers, one slice of pork roast, and one chicken thigh) is common for culinary preparation. These modeled experiment conditions created a completely different set of eleven foods that varied in many parameters. There were differences in geometric shape, in surface size, surface character (smooth, rough), in origin (plant, animal), weight, thickness and technological processing of food (with/without skin or bone), physical conditions (fresh, frozen). Evaluated samples varied also according to use marinades. The variety of shape had an effect, for example, on the tightness of the foil's adhesion to the food, and differences in the food weight affect the dispersion/dilution of the amount of aluminum transferred from the foil to the homogenized matrix. The study showed that some of the food matrices analyzed in our study are riskier in terms of aluminum transmission from the packaging foil. Samples of salmon, mackerel, and duck contained more aluminum in the homogenized matrix, although they represent different foods in terms of weight, nutritional composition, and method of preparation (Table 1 and 6).

**Table 6:** Chemical composition of marinated and not marinated samples

Samples	pH		Salt (mg/100 g)		Fat (%)		Protein (%)	
	NM	M	NM	M	NM	M	NM	M
Hermelin	5.66 ± 0.08	5.43 ± 0.12	1947.17 ± 227.30	1,334.20 ± 218.91	19.53 ± 0.99	24.09 ± 1.29	17.97 ± 0.17	11.08 ± 0.22
Tomato	3.71 ± 0.07	3.82 ± 0.02	121.14 ± 35.74	779.74 ± 69.46	0.11 ± 0.05	2.09 ± 0.42	0.54 ± 0.01	0.7 ± 0.01
Paprika	4.25 ± 0.15	4.58 ± 0.03	169.26 ± 34.82	73.16 ± 33.83	0.12 ± 0.03	0.07 ± 0.02	0.8 ± 0.00	0.76 ± 0.05
Karlovarsky knedlik	5.99 ± 0.06		1743.86 ± 85.02		0.59 ± 0.14		7.94 ± 0.06	
Pork roast	5.03 ± 0.02	5.12 ± 0.02	72.84 ± 32.96	1,304.51 ± 151.80	1.77 ± 0.12	13.43 ± 4.08	23.82 ± 0.64	20.62 ± 0.36
Pork neck	5.67 ± 0.06	5.04 ± 0.09	97.29 ± 1.68	853.00 ± 86.62	4.67 ± 1.30	7.37 ± 1.21	19.15 ± 1.05	16.88 ± 0.08
Chicken breasts	5.48 ± 0.01	5.02 ± 0.01	120.70 ± 35.45	508.36 ± 26.01	0.04 ± 0.03	0.19 ± 0.17	22.84 ± 0.36	18.99 ± 0.09
Chicken legs	5.74 ± 0.02	5.21 ± 0.03	97.33 ± 0.94	593.90 ± 64.46	10.78 ± 0.82	11.13 ± 0.30	16.09 ± 0.08	13.86 ± 0.67
Salmon	5.72 ± 0.03	5.44 ± 0.06	261.64 ± 100.42	646.94 ± 2.43	5.34 ± 0.31	12.66 ± 0.60	18.22 ± 0.71	15.43 ± 0.62
Mackerel	5.81 ± 0.01	5.49 ± 0.01	909.67 ± 7.10	1532.82 ± 0.62	0.51 ± 0.08	5.36 ± 0.27	18.52 ± 0.72	14.32 ± 0.44
Duck breasts with skin	5.47 ± 0.02	4.21 ± 0.01	86.54 ± 17.10	1,307.27 ± 323.59	18.45 ± 5.03	20.53 ± 2.21	16.06 ± 0.42	13.39 ± 0.23
Duck breasts without skin	5.57 ± 0.06	4.28 ± 0.03	184.87 ± 88.98	2034.45 ± 911.31	0.26 ± 0.14	17.03 ± 0.15	19.92 ± 0.05	16.18 ± 0.11

Abbreviations: M, marinated; NM, not marinated.

**Figure 1:**
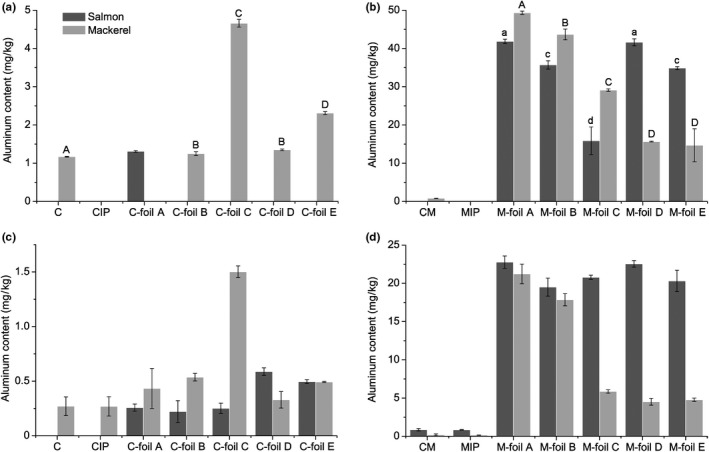
Aluminum content in baked salmon and mackerel fish (marinated and not marinated) determined by ICP‐MS and AAS. *A: aluminum content in the samples of salmon and mackerel not marinated (measured by ICP/MS), B: aluminum content in the samples of salmon and mackerel marinated (measured by ICP/MS), C: aluminum content in the samples of salmon and mackerel not marinated (measured by AAS), D: aluminum content in the samples of salmon and mackerel marinated (measured by AAS), **C: control; CIP: control packed in inert packaging; C‐foil A, C‐foil B, C‐foil C, C‐foil D, C‐foil E: samples packed in different foils CM: control marinade; MIP: samples in marinade packed in inert packaging; M‐foil A, M‐foil B, M‐foil C, M‐foil D, M‐foil E: samples in marinade packed in different foils, ***Different lowercase letters (a, b, c, d) indicate significant statistical (*p* < .05) difference between salmon samples. Different uppercase letters (A, B, C, D) indicate significant statistical (*p* < .05) difference between salmon samples

Aluminum contents in the samples of salmon and mackerel samples (both marinated and not marinated) determined by ICP‐MS and AAS are shown in Figure 1.

The aluminum contents (shown in Figure 1) measured by ICP‐MS device are showing higher measured amounts which are in accordance with literature data that ICP‐MS and AAS results can differ, and aluminum levels determined by ICP‐MS are higher. It has been found that ICP‐MS technique according to detection limits is considered an excellent method for the determination of most elements (Frankowski et al., 2011; Tyler & Jobin Yvon, 1995).

Mackerel (Scomber scombrus) fish samples without marinade showed statistically significant (*p* < .05) increase in aluminum content compared with control samples (C) (1.17 ± 0.01 mg/kg); the highest aluminum content had samples wrapped and baked in foil C (4.66 ± 0.10 mg/kg). Significant (*p* < .05) increases also occurred between marinated control samples of fish samples (salmon and mackerel) and wrapped in aluminum foils, where the highest increase was measured in mackerel samples wrapped and baked in foil A (49.34 ± 0.44 mg/kg) (Table 1).

Our results are in accordance with the results of Ertl and Goessler (2018) since they also found increment of aluminum in food (beef, fish fillet, onion, pork, poulard, and potato) during heating in aluminum foil and heat. The authors confirmed that aluminum foil side (shiny/dull) did not play an important role in aluminum leakage (Bassioni et al., 2012; Ertl & Goessler, 2018).

The exposure to aluminum by regular diet was found not to represent a risk for consumers, though experiments included the measurement of aluminum by the AAS (Muller, Anke, & Illing‐Günther, 1998; Ranau, Oehlenschläger, & Steinhart, 2001b). Our study showed that aluminum content determination by AAS was inferior in comparison with ICP‐MS measurements that revealed much higher aluminum content.

The samples of salmon and mackerel, without marinade, packed in aluminum foils during baking had lower amount of aluminum, measured by both methods (ICP‐MS and AAS). It has been indicated in previous literature data that aluminum leakage from aluminum foil is higher when food with low or high pH is in contact; higher rate of leakage was also measured in more salty food (Al Zubaidy et al., 2011).

The aluminum content increase was noticed in previous studies in canned herring fillets with tomato cream by 1,050%, after one week of storage (Ranau, Oehlenschläger, et al., 2001b). The authors monitored aluminum content during 2 years of storage and found a constant significant aluminum increase during the whole period of storage (by 1,321%). In our study, aluminum content increased by 20 times after marinating and heating of salmon and mackerel samples (Table 2). The content of aluminum increased also in cans with tomato cream (by 65%) and curry sauce (by 187%). Aluminum leakage to food packed in can occur due to the process of can sterilization, but the leakage is occurring also during can shelf life, since the prevalence of aluminum content in raw fish is under 1 mg/kg wet mass. That is the reason why aluminum content in raw seafood is considered as not a significant risk for consumers (Ranau, Oehlenschläger, et al., 2001b).

**Figure 2:**
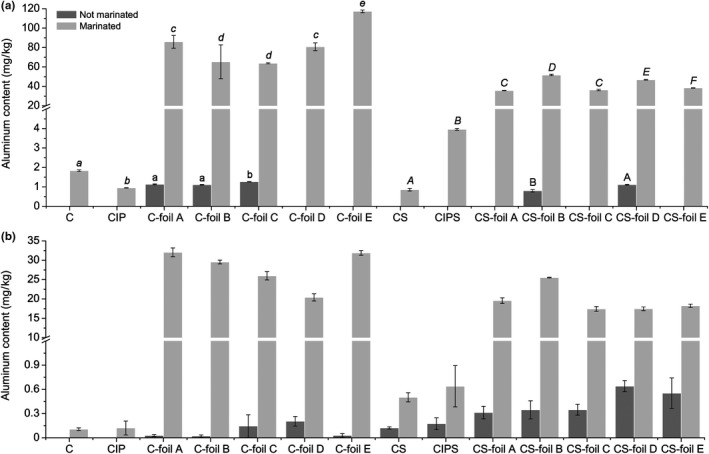
Aluminum content in baked duck breast samples (with skin and without skin) (ICP‐MS and AAS). **C: control; CIP: control packed in inert packaging; C‐foil A, C‐foil B, C‐foil C, C‐foil D, C‐foil E: samples packed in different foils CM: control marinade; MIP: samples in marinade packed in inert packaging; M‐foil A, M‐foil B, M‐foil C, M‐foil D, M‐foil E: samples in marinade packed in different foils. *A: aluminum content in the samples of duck breast without and with skin (measured by ICP‐MS). B: aluminum content in the samples of duck breast without and with skin (measured by AAS). *C: control without skin; CIP: control packed in inert packaging without skin; C‐foil A, C‐foil B, C‐foil C, C‐foil D, C‐foil E: samples packed in different foils without skin; CS: control marinade with skin; CIPS: samples in marinade packed in inert packaging with skin; CS‐foil A, CS‐foil B, CS‐foil C, CS‐foil D, CS‐foil E: samples in marinade packed in different foils with skin. ***Different lowercase letters (a, b, c) indicate significant statistical (*p* < .05) difference between duck breast samples without skin. Different uppercase letters (A, B, C) indicate significant statistical (*p* < .05) difference between duck breast samples with skin

**Figure 3:**
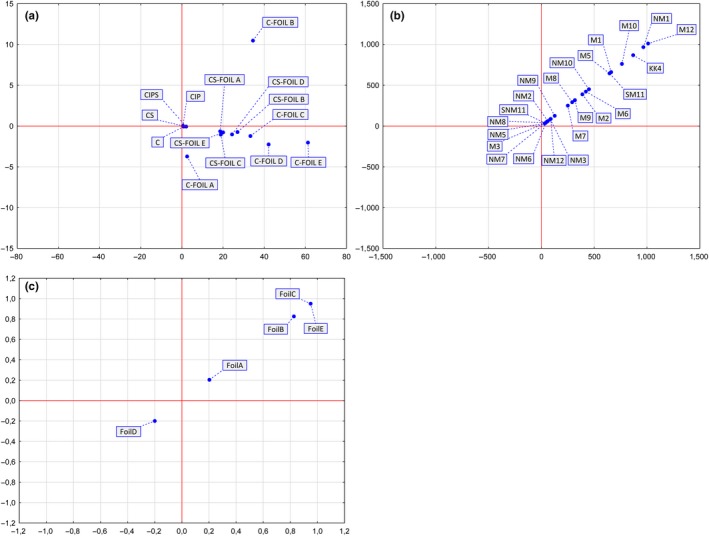
Principal component analysis of aluminum content in investigated samples and the profile of used aluminum foils. A*C: control without skin; CIP: control packed in inert packaging without skin; C‐foil A, C‐foil B, C‐foil C, C‐foil D, C‐foil E: samples packed in different foils without skin; CS: control marinade with skin; CIPS: samples in marinade packed in inert packaging with skin; CS‐foil A, CS‐foil B, CS‐foil C, CS‐foil D, CS‐foil E: samples in marinade packed in different foils with skin. B*NM1: hermelin not marinated, NM2: tomato not marinated, NM3: paprika not marinated, NM5: pork roast not marinated, NM6: pork neck not marinated, NM7: chicken breasts not marinated, NM8: chicken legs not marinated, NM9: salmon not marinated, NM10: mackerel not marinated, NM12: duck breasts without skin not marinated. M1: hermelin marinated, M2: tomato marinated, M3: paprika marinated, M6: pork neck marinated, M7: chicken breasts marinated, M8: chicken legs marinated, M9: salmon marinated, M10: mackerel marinated, M12: duck breasts without skin marinated. KK4: karlovarsky knedlik, SNM11: duck breasts with skin not marinated, SM11: duck breasts with skin marinated

Aluminum contents in duck breasts wrapped in aluminum foil and baked are shown in Figure 2 and Table 2. Not marinated samples had very low increment of aluminum contents (the samples wrapped in foil C: 0.61 ± 0.01 mg/kg wet mass), but marinated samples showed significantly (*p* < .05) higher increment (the samples without skin wrapped in foil E: 45.18 ± 0.01 mg/kg wet mass). Statistically significant (*p* < .05) higher aluminum contents were measured in the samples without skin in comparison to samples with skin (the samples with skin wrapped in foil B: 22.79 ± 0.13) (Table 2, Figure 3).

According to these results, we have come to the new conclusion that, in addition to the known facts previously published, skin as a natural barrier has a major effect on aluminum transmission. We assume that all three skin layers (epidermis, dermis, and subcutis) have barrier properties and their components such as protein fibers, especially keratin, collagen, and elastin have direct responsibility for the aluminum's impermeability into the inner layers of the food. Similar behavior can be assumed in matrices with different intramuscular tissue content, that is, aluminum will be present in larger quantities in deeper layers of fish meat (which have a very low content of stroma proteins) compared to mammalian or poultry meat.

It was found that during baking of food wrapped in aluminum foil at lower temperatures (<160ºC), the leakage of aluminum is at a lower rate than when the process of baking is done at temperatures over 220ºC (Turhan, 2006). Previous studies indicate that cooking temperature is more influencing aluminum leaching than cooking time, due to the changes of oxide layer from an amorphous to a crystalline structure. Oppositely, from our results it had been stated that aluminum foil is resistant to corrosion in the pH range of 4–8.5 (Lamberti & Escher, 2007).

Aluminum contents in the samples of marinated duck breast, the both marinated and not marinated, without skin and with skin are shown in Figure 3, representing principal component analysis (PCA). PCA is showing highly significant differences in aluminum content levels between analyzed samples of duck breasts; showing statistically significant (*p* < .05) differences among samples baked and wrapped in different foils, especially differences (*p* < .05), are noticeable between samples without and with skin (Figure 3).

The occurrence of aluminum ions in food and especially secondary food contamination with aluminum represents the risk for consumers also since elements such as zinc, magnesium, and iron are essential for organism, and no scientific studies have indicated that aluminum plays an important role for a living organism (Schafer & Seifert, 2006; Stahl et al., 2018). Oppositely, aluminum intake represents a health risk, affecting hazardously nervous system, bones, and hemopoietic system (Becaria, Campbell, & Bondy, 2002).

**Figure 4:**
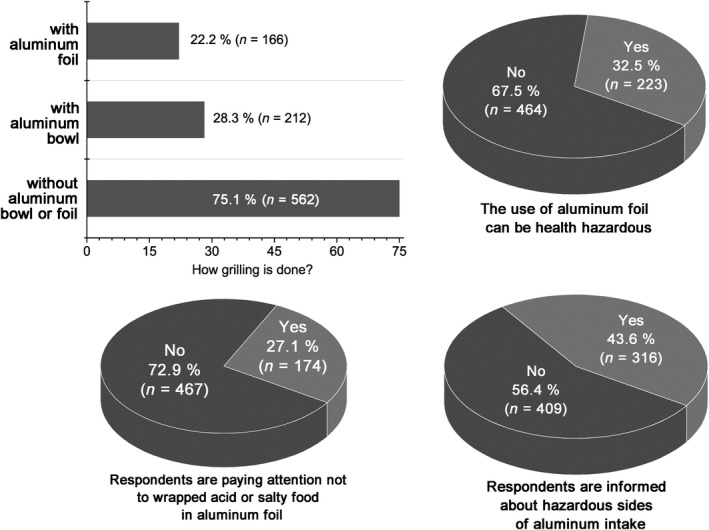
Respondents’ preferences concerning the use of aluminum foil

The compositions of aluminum foils used in the research are presented with principal component analysis (PCA) (Figure 3). As it can be seen in Figures 3 and 4, groups are formed out of used aluminum foils compositions. This finding is showing how aluminum foils that are present on the market distinguished between themselves statistically significant (*p* < .05). The inclusion of other metals in aluminum foils affects its corrosion. Magnesium is often incorporated in aluminum foil due to the mechanical strength improvement, though higher magnesium content leads to lower stability of the alloy against weak acids (Lamberti & Escher, 2007). Though, older literature data stressed out that the leakage of aluminum from aluminum foil is almost neglectable in following food types and products: starch, sugar, egg, powder, coffee, chocolate, and tea packed in bare aluminum foil and undergone treatments such as storing at room temperature, in the refrigerator, freezing, and heating (Kunze, 1976; Lamberti & Escher, 2007; Servus, 1989). The neglectable leakage of aluminum was also found in the studies that included acidic beverages in coated aluminum cans and white wines in aluminum cans (Bloeck, Kreis, & Stanek, 1986; Dürr & Bloeck, 1987).

The significant (*p* < .05) differences between marinated and not marinated food samples, included in the research, are confirming findings that aluminum dissolution is dependent on pH, same as on temperature (Bi, 1996). Bi (1996) explained that aluminum leaching is happening according to the following formula:

$${\text{Al}}_{2}O_{3}+{\text{6H}}^{+}={\text{2Al}}^{3+}{\;\text{+ 3H}}_{2}O$$

Aluminum ions in food react with organic acids and the degree of aluminum leakaging depends on food composition (Al Juhaiman, 2010; Scancar, Stibilj, & Miacic, 2004).

Aluminum leaching from aluminum foil represents an existent problem/issue, especially because aluminum leaching can be increased during culinary preparation, people may lower acidity or add salt, known agents that increase the transfer level of aluminum from aluminum foil. Oppositely, sugar added to food during heating in aluminum foil reduces aluminum leaching due to the formation of kind of coating (Joshi, Toma, Medora, & O’Connor, 2003; Verissimo et al., 2006).

Aluminum contents in other investigated food samples (hermelin, tomato, paprika, karlovarsky knedlik, pork roast, pork neck, chicken breasts, and chicken thighs) are present in Table 4. The highest concentration of aluminum was measured in marinated pork roast (6.90 ± 0.08 mg/kg wet mass). These samples were marinated and wrapped in foil E, the foil on which labeling was written that it can be used for the baking. These concentrations can be considered low, but they are higher in comparison with the results of Ertl and Goessler, (2018), and their aluminum levels in pork and fish fillets samples were under 1 mg/kg. Though, they found increment up to 4 times compared with raw samples. The lower aluminum contents in the study of Ertl and Goessler, (2018), probably, occurred due to lower baking temperatures (180ºC) than temperatures applied in our study (220°C). Our aluminum level findings are much lower (under 6 mg/kg dry mass) in chicken breast and thighs samples in comparison with the study of Turhan (2006) whose aluminum levels were around 50 mg/kg dry mass (under the following conditions: baked 20 min at 250°C).

The hazardous side of aluminum foil used for food preparation and storage was also emphasized by the finding that aluminum content in food was increased during 1 to 3 days of storage in aluminum foil. The aluminum content was higher than 60 mg/kg and more than 20 mg/kg in ham and cheese samples stored for 3 days in aluminum foil, respectively (Ertl & Goessler, 2018).

Chemical composition of investigated samples is shown in Table 6. PCA analysis is emphasizing that there is no significant difference (*p* < .05) between samples’ chemical compositions (Figure 3). Found aluminum contents are not alarming, but the estimated dietary exposure to aluminum in various countries is between 1.3 mg/day to 13 mg/day, while in European countries it is from 1.6 to 13 mg/day (Bratakos et al., 2012). These data are indicating that dietary aluminum exposure is from 0.2 to 1.5 mg/per kg of body mass in a 60 kg adult (EFSA, 2008).

### 3.1 Survey

Humans are exposed to aluminum mainly through diet, meaning that consumers’ sociodemographic attributes, eating habits, same as culinary preparation play the important role in aluminum daily intake (Yokel, 2012). Aluminum intake through daily diet is obviously influenced by consumers’ preferences. Respondents’ preferences toward the use of aluminum foil for food preparation are shown in Figure 4.

The issue can be overviewed through the results gained by the survey (Figure 4). The majority of respondents (72.9%; *n* = 467) answered that they do not check the labeling of aluminum foil, meaning they do not read the information that usually food should not be heated wrapped in aluminum foil, especially sour and salty food. Respondents’ current status influenced significantly (*p* < .05) the checking of aluminum foil labeling (unemployed respondents and an employee of state checked aluminum foil less often) (Figure 4).

The majority of respondents (67.5%; *n* = 464) stated that they had not been looking for the information about the hazardous side of aluminum content in food,and they (56.4; *n* = 409) did not know how the intake of aluminum can adversely affect human organisms (Figure 4). Chi‐square test showed that women, married respondents, respondents under 20 years, and older than 30 ages are statistically significant (*p* < .05) more aware of aluminum use hazardous sides, in food preparation, while education level did not affect respondents’ preferences toward the use of aluminum foil.

The important aspect of the survey is how respondents are informed about hazardous sides of aluminum usage. It was found that statistically significant (*p* < .05) that women, married respondents, and respondents aging from 31 to 50 years are better informed about hazardous sides of aluminum usage (Figure 4).

## 4 CONCLUSION

The study is showing leakage of aluminum to food that was baked in aluminum foil, and the research is indicating that excessive consumption of food prepared by baking in aluminum foil can carry a health risk. Although the measured level of aluminum contamination in food used in the experiment is not alarming, it is probably hard to achieve 2 mg/kg of body mass weekly limit, but due to hazardous side of aluminum intake it can have high potential risk to people with certain aliments (especially people suffering from chronic renal failure) and smaller children. Beside these statements, aluminum absorption is well influenced by the presence of elements as iron, calcium, zinc, same as additive such as citrate (Al Juhaiman, 2010; Verrissimo et al., 2006). Our data will certainly serve as useful source and confirmation of the hazardous side of aluminum foil use during food culinary preparation, same as the confirmation of not so predictable aluminum contamination in different food types. The survey also confirmed that consumers are not enough informed about hazardous side of aluminum foil usage.

In conclusion, we can say the target of the study was met, and we have gained new up‐to‐date information on aluminum levels in a wide range of foods that was not been available in this research area. Certainly that this pilot experiment will be used for more detailed case studies in future.

## CONFLICTS OF INTEREST

The authors declare no conflict of interest or relationship, financial or otherwise.

## ETHICAL APPROVAL

This study does not involve any human testing.

## References

[fsn31204-bib-0001] Al Juhaiman, L. A. (2010). Estimating Aluminum leaching from Aluminum cook wares in different meat extracts and milk. Journal of Saudi Chemical Society, 14, 131–137. 10.1016/j.jscs.2009.12.020

[fsn31204-bib-0002] Al Juhaiman, L. A. , Al‐Shihry, R. A. , & Al‐Hazimi, H. M. (2014). Effect of cardamom extract on leaching of aluminum cookware. International Journal of Electrochemical Science, 9, 1055–1070.

[fsn31204-bib-0003] Al Zubaidy, E. A. H. , Mohammad, F. S. , & Bassioni, G. (2011). Effects of pH, salinity and temperature on aluminum cookware leaching during food preparation. International Journal of Electrochemical Science, 6, 6424–6441.

[fsn31204-bib-0004] Bassioni, G. , Mohammed, F. S. , Al Zubaidy, E. , & Kobrsi, I. (2012). Risk assessment of using aluminum foil in food preparation. International Journal of Electrochemical Science, 7, 4498–4509.

[fsn31204-bib-0005] Becaria, A. , Campbell, A. , & Bondy, S. C. (2002). Aluminium is a toxicant. Toxicology and Industrial Health, 18, 309–320. 15068131 10.1191/0748233702th157oa

[fsn31204-bib-0006] Bi, S. (1996). A Model describing the complexing effect on leaching of aluminum from cooking utensils. Environmental Pollution, 92, 85–89. 15091415 10.1016/0269-7491(95)00038-0

[fsn31204-bib-0007] Bloeck, S. , Kreis, A. , & Stanek, O. (1986). Vergleichende bestimmung von aldehyden und ketonen in apfelsaft nach 2 jähriger lagerung in innengeschützten aluminium‐und weissblechdosen bei 4 und 20°C. Alimenta, 25, 23–28.

[fsn31204-bib-0009] Bratakos, S. M. , Lazou, A. E. , Bratakos, M. S. , & Lazos, E. S. (2012). Aluminium in food and daily dietary intake estimate in Greece. Food Additives and Contaminants: Part B, 5, 33–44. 10.1080/19393210.2012.656289 24779693

[fsn31204-bib-0010] Center for Food Safety (2009). *Risk assessment studies Report No. 35: Chemical hazard evaluation: Aluminum in food* . The Government of the Hong Kong Special Administrative Region, Hong Kong.

[fsn31204-bib-0011] Darbre, P. D. , Pugazhendhi, D. , & Mannello, F. (2011). Aluminium and human breast diseases. Journal of Inorganic Biochemistry, 105, 1484–1488. 10.1016/j.jinorgbio.2011.07.017 22099158

[fsn31204-bib-0012] Dürr, P. , & Bloeck, S. (1987). Zum Verhalten von Weisswein in Kleinflaschen und Aluminiumdosen. Deutsche Lebensmittel‐Rundschau, 83, 385–389.

[fsn31204-bib-0013] EAFA European Aluminium Foil Association (2016). Alufoil statistics 2015: European aluminium foil production ends 2015 with positive mood. Düsseldorf.

[fsn31204-bib-0014] EFSA (2008). Scientific opinion of the panel on food additives, flavourings, processing aids and food contact materials on a request from European commission on safety of aluminium from dietary intake. EFSA Journal, 754, 1–34. 10.2903/j.efsa.2008.754 PMC10193631 37213837

[fsn31204-bib-0015] EFSA (2011). On the Evaluation of a new study related to the bioavailability of aluminium in food. EFSA Journal, 9, 1–16. 10.2903/j.efsa.2011.2157 PMC13177353 42148405

[fsn31204-bib-0016] Ertl, K. , & Goessler, W. (2018). Aluminium in foodstuff and the influence of aluminium foil used for food preparation or short time storage. Food Additives & Contaminants: Part B, 11, 153–159. 10.1080/19393210.2018.1442881 29486656

[fsn31204-bib-0017] Fekete, V. , Vandevijvere, S. , Bolle, F. , & Van Loco, J. (2013). Estimation of dietary aluminium exposure of the Belgian adult population: Evaluation of contribution of food and kitchenware. Food and Chemical Toxicology, 55, 602–608. 23402858 10.1016/j.fct.2013.01.059

[fsn31204-bib-0018] Frankowski, M. , Zioła‐Frankowska, A. , Kurzyca, I. , Novotný, K. , Vaculovič, T. , Kanický, V. , … Siepak, J. (2011). Determination of aluminium in groundwater samples by GF‐AAS, ICP‐AES, ICP‐MS and modelling of inorganic aluminium complexes. Environmental Monitoring and Assessment, 182, 71–84. 10.1007/s10661-010-1859-8 21274747

[fsn31204-bib-0019] Gitelman, H. (1989). Aluminum and health. New York, NY: Marcel Dekker Inc.

[fsn31204-bib-0020] González‐Weller, D. , Gutiérrez, Á. , Rubio, C. , Revert, C. , & Hardisson, A. (2010). Dietary intake of aluminum in a Spanish population (Canary Islands). Journal of Agricultural and Food Chemistry, 58, 10452–10457. 10.1021/jf102779t 20809646

[fsn31204-bib-0021] Joshi, S. P. , Toma, R. B. , Medora, N. , & O’Connor, K. (2003). Detection of Aluminum in sauces packaged in Aluminum pouches. Food Chemistry, 83, 383–386.

[fsn31204-bib-0022] Kim, M. S. , & Clesceri, L. S. (2001). Aluminum exposure: A study of an effect on cellular growth rate. Science of the Total Environment, 278, 127–135. 10.1016/S0048-9697(00)00892-5 11669261

[fsn31204-bib-0023] Kunze, E. (1976). Korrosivität Verschiedener Lebensmittelgruppen Gegenüber Packmittel aus Aluminium. Aluminium, 52, 296–301.

[fsn31204-bib-0024] Lamberti, M. , & Escher, F. (2007). Aluminium foil as a food packaging material in comparison with other materials. Food Reviews International, 23, 407–433. 10.1080/87559120701593830

[fsn31204-bib-0025] Ma, N. , Liu, Z.‐P. , Yang, D.‐J. , Liang, J. , Zhu, J.‐H. , Xu, H.‐B. , … Li, N. (2016). Risk assessment of dietary exposure to aluminium in the Chinese population. Food Additives and Contaminants Part A, 33, 1557–1562. 10.1080/19440049.2016.1228125 27595294

[fsn31204-bib-0026] Müller, M. , Anke, M. , & Illing‐Günther, H. (1998). Aluminium in foodstuffs. Food Chemistry, 61, 419–428. 10.1016/S0308-8146(97)00085-X

[fsn31204-bib-0028] Ranau, R. , Oehlenschläger, J. , & Steinhart, H. (2001b). Aluminium content in edible parts of seafood. European Food Research and Technology, 212, 431–438. 10.1007/s002170000283

[fsn31204-bib-0029] Rittirong, A. , & Saenboonruang, K. (2018). Quantification of aluminum and heavy metal contents in cooked rice samples from Thailand markets using inductively coupled plasma mass spectrometry (ICP‐MS) and potential health risk assessment. Emirates Journal of Food and Agriculture, 30, 372–380.

[fsn31204-bib-0030] Scancar, J. , Stibilj, V. , & Miacic, R. (2004). Determination of aluminum in Slovenian foodstuffs and its leachability from Aluminum cookware. Food Chemistry, 85, 151–157.

[fsn31204-bib-0031] Schäfer, U. , & Seifert, M. (2006). Oral intake of aluminium from foodstuffs, food additives, food packaging, cookware and pharmaceutical preparations with respect to dietary regulations. Trace Elem Elektroly, 23, 150–161.

[fsn31204-bib-0032] Severus, H. (1989). The use of aluminium — Especially as packaging material — in the food Industry In Massey R., & Taylor D. (Eds.), Aluminium in food and the environment (pp. 88–101). Cambridge, UK: Royal Society of Chemistry, 73.

[fsn31204-bib-0033] Stahl, T. , Falk, S. , Taschan, H. , Boschek, B. , & Brunn, H. (2018). Evaluation of human exposure to aluminum from food and food contact materials. European Food Research and Technology, 244, 1–8. 10.1007/s00217-018-3124-2

[fsn31204-bib-0034] Turhan, S. (2006). Aluminium contents in baked meats wrapped in aluminium foil. Meat Science, 74, 644–647. 10.1016/j.meatsci.2006.03.031 22063217

[fsn31204-bib-0035] Tyler, G. , & Jobin Yvon, S. (1995). *ICP‐OES, ICP‐MS and AAS techniques compared* . ICP Optical Emission Spectroscopy Technical Note, 5.

[fsn31204-bib-0036] Verissimo, M. I. S. , Oliveira, J. , & Gomes, M. (2006). Leaching of Aluminum from cooking pans and food containers. Sensors and Actuators B, 118, 192–197.

[fsn31204-bib-0037] World Health Organization, 1989World Health Organization (1989). *FAO/WHO expert committee report on food additives: 33rd report. WHO Technical Report Series* . Geneva, Switzerland: World Health Organization.

[fsn31204-bib-0038] World Health Organization, 2011World Health Organization (2011). *FAO/WHO expert committee report on food additives: 47rd report. WHO Technical Report Series* . Geneva, Switzerland: World Health Organization.

[fsn31204-bib-0039] Yokel, R. A. (2012). *Aluminum in food–the nature and contribution of food additives* . InTech: In Food Additive.

